# Left Jugular Approach Predisposes to Malposition of Hemodialysis Catheter Into the Azygos Vein

**DOI:** 10.7759/cureus.90601

**Published:** 2025-08-20

**Authors:** Chung-Wai Wong, Hong-Ching Lee

**Affiliations:** 1 Department of Radiology and Organ Imaging, United Christian Hospital, Kowloon, HKG

**Keywords:** azygos vein, case report, catheter complication, catheter malposition, central venous catheter, computed tomography, hemodialysis, interventional radiology, left internal jugular vein, vascular access

## Abstract

Malposition of central venous catheters (CVCs) into the azygos vein is an uncommon but significant complication. The left internal jugular vein (IJV) approach is a risk factor for this malposition. To our knowledge, only two published cases of azygos malposition of left jugular CVC had corresponding computed tomography (CT) images. This report uses novel CT reformats to illustrate why left IJV access may predispose to azygos catheterization. We present the case of a 68-year-old male with end-stage renal failure requiring hemodialysis (HD). Due to right IJV stenosis, a tunneled HD catheter was inserted via the left IJV. A post-procedure chest radiograph revealed the catheter tip malpositioned in the azygos vein, exhibiting a characteristic "fishhook" appearance, which CT subsequently confirmed. The catheter was successfully repositioned. CT reformats provided a clear anatomical explanation, demonstrating how the oblique trajectory of the left brachiocephalic vein relative to the superior vena cava and the posterior location of the azygos ostium predisposed the catheter to enter the azygos vein. This case underscores the importance of recognizing radiographic signs and understanding the anatomical challenges associated with a left-sided CVC approach. The left IJV approach for CVC placement carries an inherent risk of azygos malposition. Vigilant radiographic assessment is crucial, and the use of routine fluoroscopy should be strongly considered for this approach.

## Introduction

A tunneled hemodialysis (HD) catheter is a type of central venous catheter (CVC) commonly placed in patients requiring dialysis for extended periods while awaiting an arteriovenous fistula or graft, or if such options are not feasible. The ideal tip position for a tunneled HD catheter is the mid-right atrium [1]. In other neck or upper limb CVCs, the tip is normally placed at the superior vena cava (SVC) or cavoatrial junction. However, malposition into the azygos vein can rarely occur [2]. This is clinically significant, as azygos malposition may lead to complications including catheter malfunction, venous perforation, venous thrombosis, back pain, and tracheo-azygos fistula [3,4]. In contrast to the preferred right internal jugular approach, the left internal jugular approach is a risk factor for azygos malposition [2,5]. Image-based analysis of how azygos malposition occurs in the left jugular approach has been limited. Only two published cases of the azygos malposition of a CVC via a left jugular approach had corresponding computed tomography (CT) images, to the best of our knowledge [5,6]. In the following case, we delineate why left jugular access may predispose to azygos vein catheterization, using multiplanar reformatted CT images.

## Case presentation

A 68-year-old gentleman had end-stage renal failure due to cast nephropathy resulting from Kappa light chain myeloma. After three years of peritoneal dialysis (PD), he had to switch to HD due to encapsulating peritoneal sclerosis, causing PD failure.

**Figure 1:**
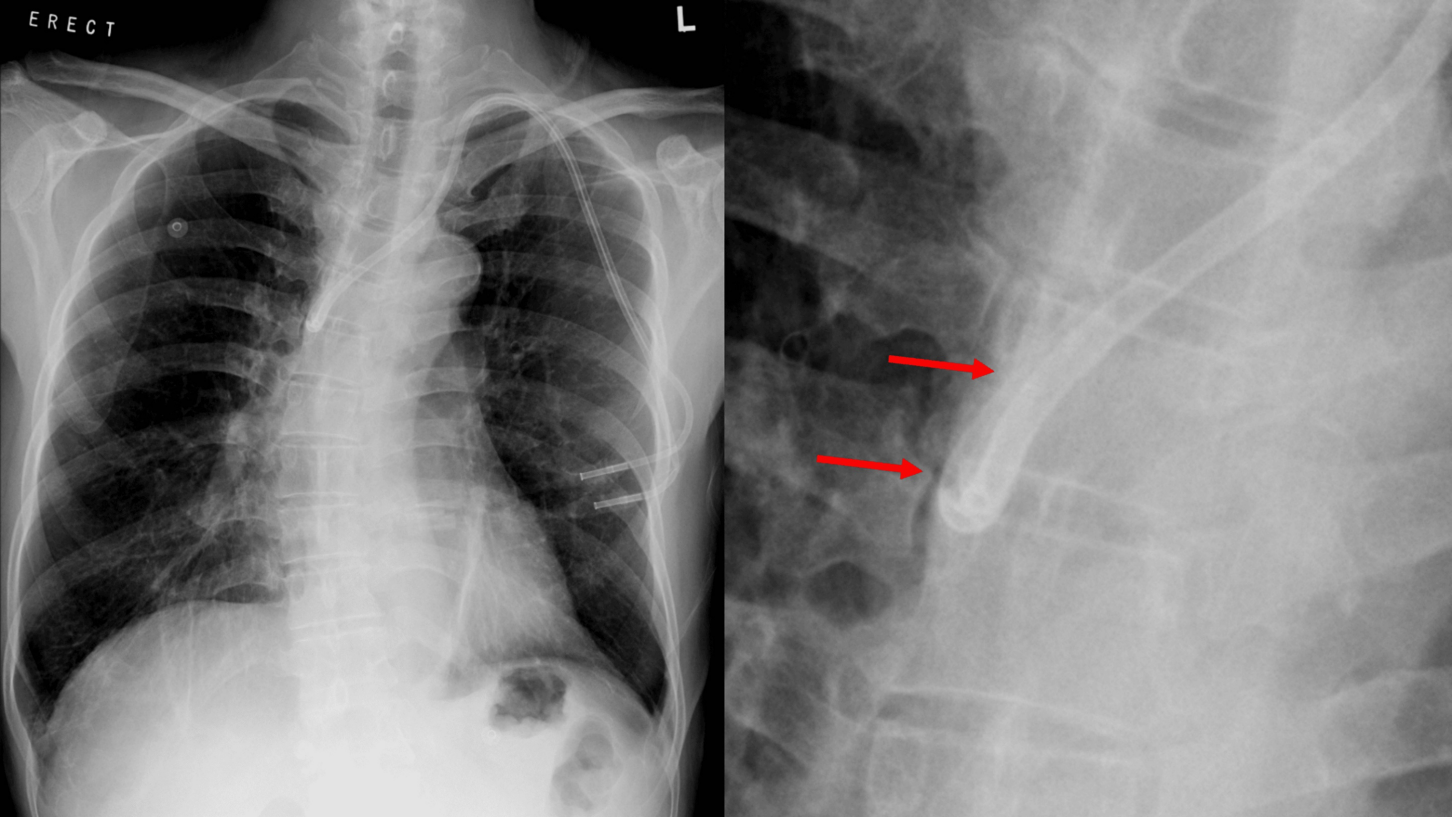
CXR (right) and zoomed in at the catheter tip (left) A left jugular tunneled split-stream CVC is noted. Both tips of the catheter (arrows) are superimposed at the right tracheobronchial angle, with an abnormal fishhook appearance. These findings are highly suggestive of catheter tip malposition into the azygos vein. CVC: malposition of central venous catheter, CXR: chest radiograph

**Figure 2:**
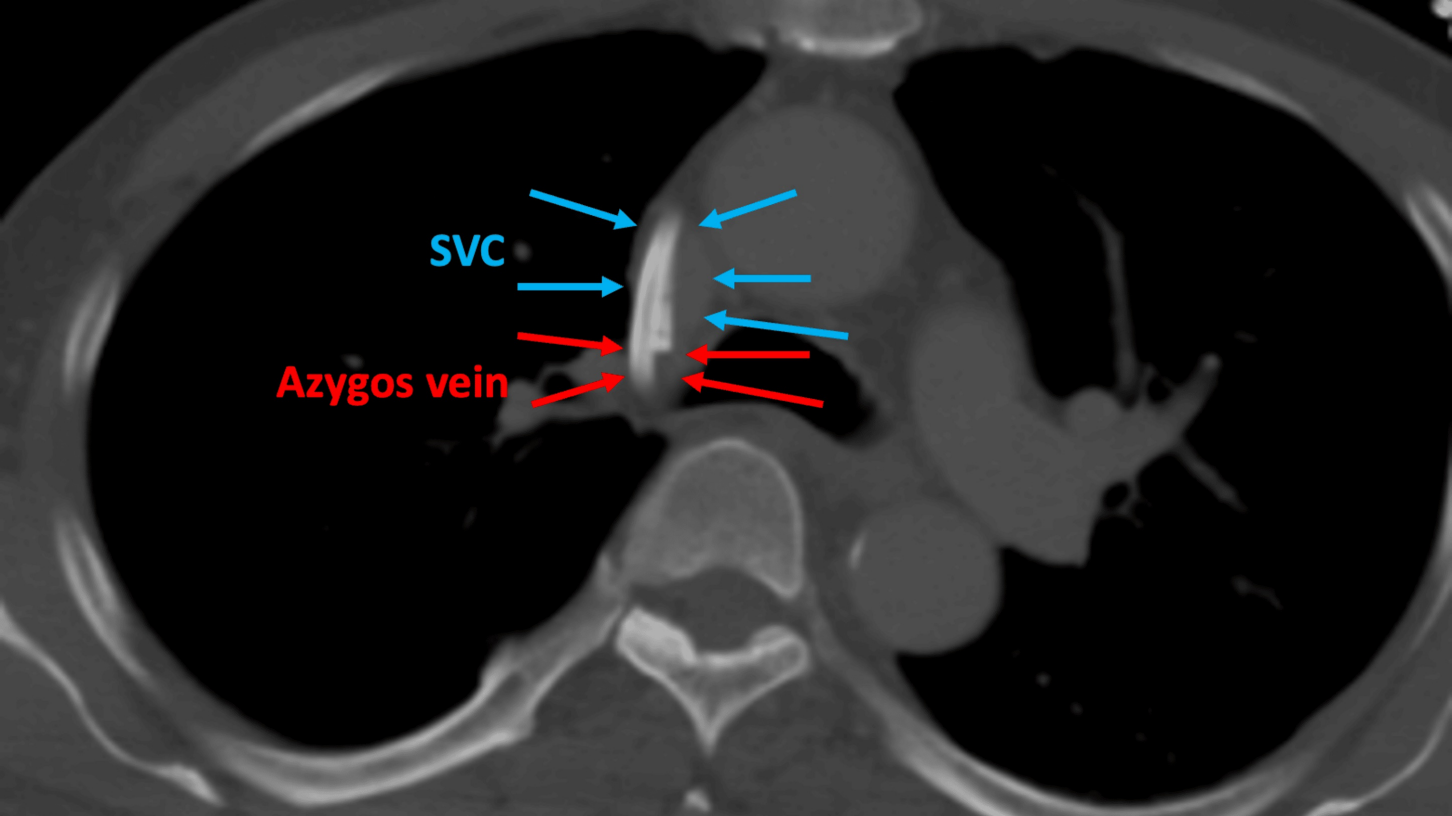
Contrast CT thorax axial slice Confirmed malposition of both catheter tips into the azygos vein (red arrows). Blue arrows denote SVC. CT: computed tomography, SVC: malposition of central venous catheter

Due to a stenosed right internal jugular vein (IJV), initial HD access was established via a left IJV approach. A 13 cm dual-lumen acute (non-tunneled) HD catheter was inserted into the left IJV under ultrasound guidance using the Seldinger technique. Three weeks later, this was converted to long-term access. A guidewire was inserted through the existing acute catheter, and a 32 cm tunneled split-stream HD catheter was then placed via the left IJV using a peel-away sheath after serial dilation. The catheter was tunneled to an exit site below the clavicle. The catheter was patent with free flow of blood from both limbs at the end of the procedure. Following catheter insertion, the patient remained asymptomatic. A confirmatory radiograph was performed after the procedure without the use of intra-procedural fluoroscopic guidance. A post-procedure chest radiograph (CXR) showed that both tips of the split-stream catheter were superimposed at the right tracheobronchial angle, with an abnormal fishhook appearance, which was highly suggestive of catheter tip malposition into the azygos vein (Figure 1). Subsequent contrast-enhanced CT of the thorax confirmed malposition of both catheter tips into the azygos vein (Figure 2).

A new catheter was placed using the guidewire exchange technique via the existing catheter under fluoroscopic guidance, and satisfactory positioning was achieved. No other catheter complications were found other than the need for repositioning. Three months later, a right brachiocephalic fistula was eventually created, alleviating the need for the HD catheter.

## Discussion

The right IJV approach is preferred for HD catheters according to the Kidney Disease Outcomes Quality Initiative (KDOQI) guideline. It would result in a more direct path into the SVC via the right brachiocephalic vein (BCV) and has lower complication rates. If the right IJV approach is contraindicated (e.g., stenosis), an alternative approach including the left IJV may be needed [1].

A left jugular approach was found to be a risk factor for azygos malposition as compared to the conventional right jugular access, among 16 likely cases of azygos malposition out of 1287 CVCs [2]. In that series, the diagnoses were based on purely radiographic clues from retrospective review of post-procedure or follow-up radiographs. This was later supported by another retrospective image review of 11 azygos malpositions of CVCs [5]. In another, more recent article, left and right jugular approaches each accounted for 50% of 16 reported cases [7]. The authors concluded that left or right jugular approaches have “similar susceptibility.” However, since the left jugular approach is less commonly performed, there may be an over-representation of risk per attempt with the left jugular approach.

Azygos malposition can be a direct sequel of catheter insertion or result from later catheter migration [2,5]. Clinically, this may manifest as catheter dysfunction (e.g., inability to aspirate blood), chest or back pain, or, in rare cases, severe complications like venous perforation. However, the presentation is often clinically silent, as was the situation in our patient, underscoring the unreliability of clinical signs alone [3]. Other causes for azygos malposition include dilation of the azygos vein, high central venous pressure, and blockage of the IVC or SVC [3].

The azygos vein begins in the abdomen as the ascending lumbar vein and passes through the aortic hiatus into the thorax. After ascending along the right side of the vertebral column, it arches anteriorly and enters the SVC at its posterior wall at the T4-T5 level.

It is important to detect azygos malposition of a CVC on CXR. On a frontal CXR, a kink or fishhook appearance is often seen near the right tracheobronchial angle, where the azygos vein arches over the right main bronchus to enter the SVC [6], which was seen in our case. Suppose the catheter progresses further caudally within the azygos vein. In that case, a medial deviation of the catheter tip across the midline spinous processes can be seen, corresponding to the course of the azygos vein. A lateral CXR could demonstrate the catheter extending posteriorly from the SVC and forming an upward convexity [2]. Malposition into the right internal thoracic vein may present a similar appearance on a frontal radiograph, with the tip in the right paramediastinal region just lateral to the SVC; however, a lateral CXR can confidently differentiate between the two, as this catheter would course anteriorly [8]. Since the azygos vein projects over the SVC on a frontal CXR, diagnosing azygos malposition can be less straightforward, and in such cases, a venogram performed via the CVC is a practical way to confirm the malposition. In patients with CVC obstruction, chest discomfort, or pleural effusion, it is recommended to perform an enhanced chest CT to exclude vascular perforation [9].

During fluoroscopic guidance of CVC insertion, a straight vertical course of the guidewire advancing well below the diaphragm is a reliable indicator of correct placement within the vena cava. Conversely, an abnormal kink at the right tracheobronchial angle is a significant red flag for azygos placement. This sign remains a concern even if the guidewire tip reaches just past the diaphragm, since the azygos vein originates from abdominal tributaries [10]. A useful tactile clue can also help differentiate the two: the J-tip guidewire should spin freely within the capacious vena cava, whereas resistance may be felt if the tip is lodged in the narrower azygos vein.

**Figure 3:**
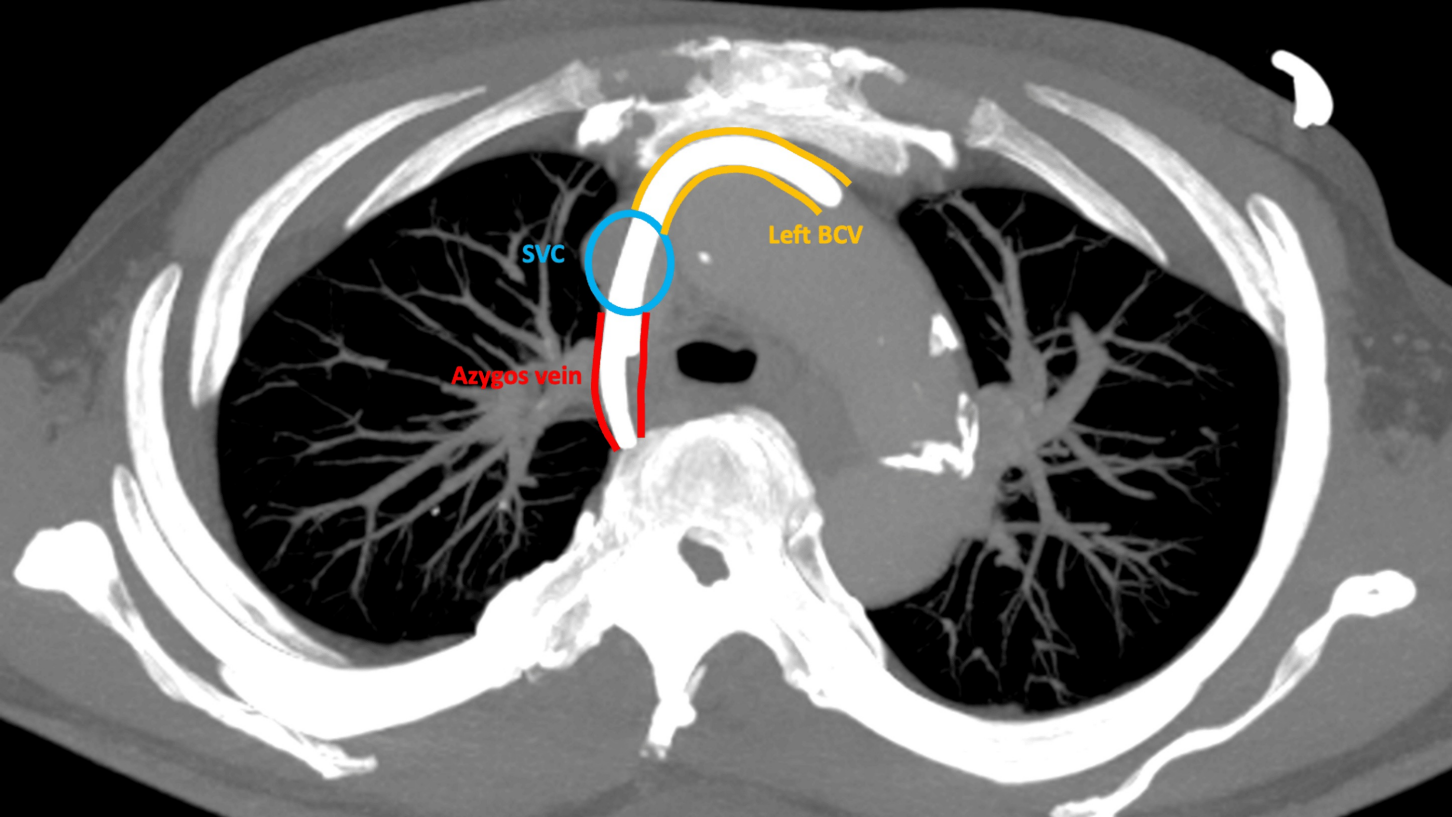
Contrast CT thorax of this case: MIP image in the axial plane The final portion of the left BCV (yellow vessel), where it meets the SVC (blue lumen), has an anterior-to-posterior trajectory. The catheter therefore preferentially entered the azygos ostium located on the posterior wall of the SVC, followed the azygos vein (red vessel), and traveled further posteriorly. MIP: maximum intensity projection, CT: computed tomography, BCV: brachiocephalic vein, SVC: malposition of central venous catheter

Our CT reformats show how a left jugular access predisposes to azygos malposition. In the axial plane, the final portion of the left BCV has an anterior to posterior trajectory where it meets the SVC (Figure 3). The catheter or guidewire within the left BCV is more likely to encounter the azygos opening posteriorly in the SVC, allowing it to travel posteriorly into the azygos arch. This is in contrast with the right BCV, which joins the SVC without such a significant anterior-posterior trajectory.

**Figure 4:**
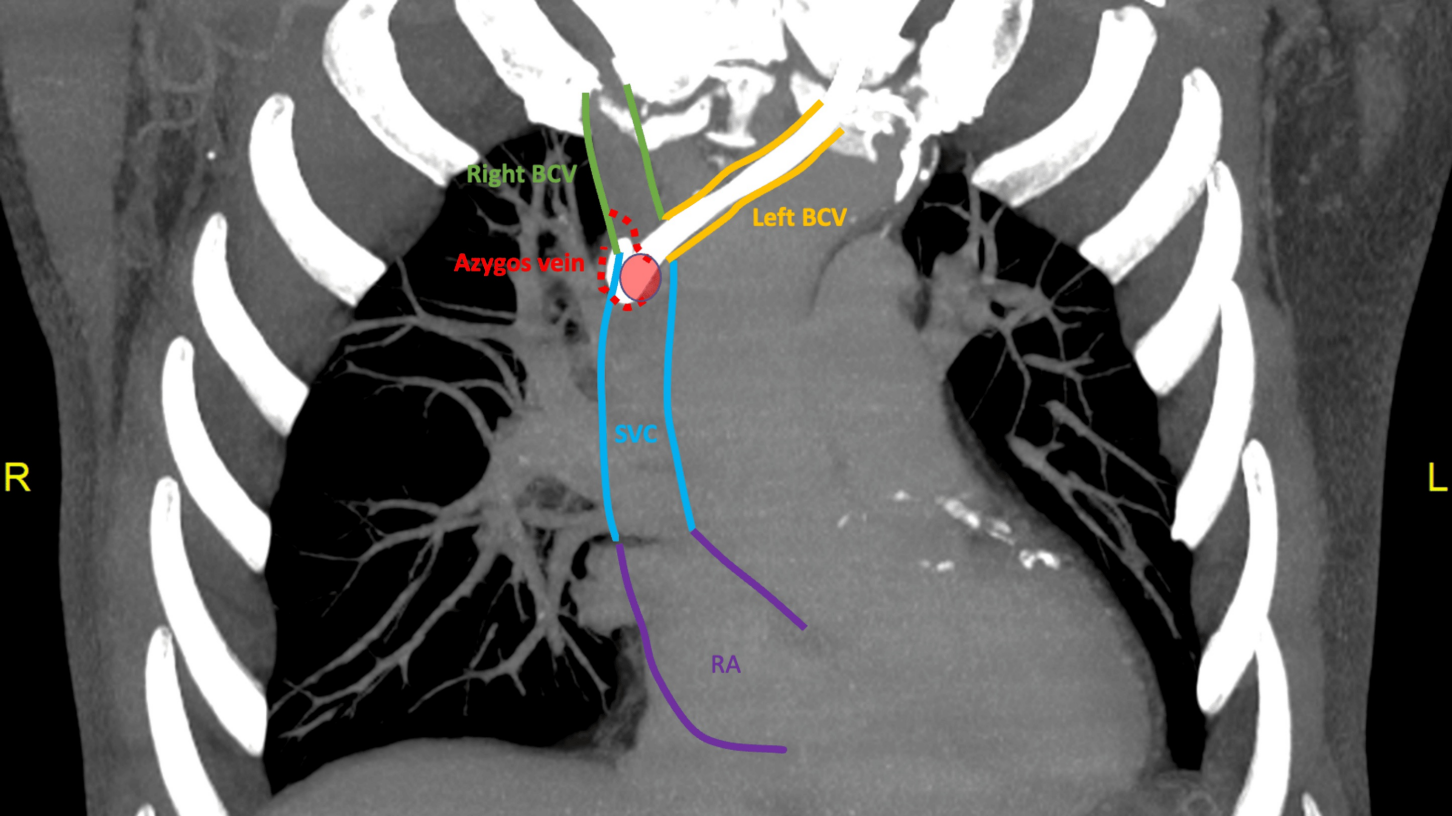
Contrast CT thorax of this case: MIP image in the coronal plane The red circle denotes the azygos opening on the posterior wall of the SVC. The red dotted line shows the course of the azygos vein out of the image plane. The CVC within the left BCV (yellow vessel) meets the SVC (blue vessel) in a more oblique fashion when compared to the right BCV (green vessel). This oblique entry requires the catheter to make a more acute turn to follow the SVC's vertical path; thus, it may inadvertently follow the relatively horizontal course of the azygos vein. The catheter tip within the azygos vein, therefore, gives rise to the fishhook appearance when projected on a frontal radiograph. The purple lines outline the right atrium. MIP: maximum intensity projection, CT: computed tomography, BCV: brachiocephalic vein, SVC: malposition of central venous catheter

In the coronal plane, the left BCV joins the SVC in an oblique fashion; therefore, the catheter or guidewire needs to make a more acute turn before it can follow the vertical downward path of the SVC (Figure 4). It, therefore, may inadvertently follow the relatively horizontal course of the azygos vein. This is in contrast with the right BCV, which joins the SVC much more vertically.

Given that the left jugular approach is associated with more risk, routine fluoroscopy should be utilized during CVC insertions requiring this approach. It is important to note that smooth aspiration or injection via the catheter does not exclude malposition [10], which was also observed in our case.

The management of azygos malposition is repositioning under radiological guidance. This case demonstrated successful salvage of the same access site using the guidewire exchange technique. Some authors have also reported successful repositioning of the CVC by carefully withdrawing the CVC away from the azygos vein by the corresponding length [9]. A history of azygos malposition predisposes to later migration [5], justifying a lower threshold for re-imaging upon any catheter dysfunction.

## Conclusions

It is important to detect azygos vein malposition of a CVC on radiographs, as it may be clinically silent, as in our patient. However, it may subsequently lead to severe complications in some cases. The reformatted CT images we presented provide insight into how a left jugular approach predisposes to azygos vein malposition. Interventionalists should exercise extra caution when a left jugular approach is required for CVCs, which includes the use of routine fluoroscopy.
